# Supplementary material for: Evidence for endogenous hydrogen peroxide production by E. coli fatty acyl-CoA dehydrogenase
Source: PLoS One. 2024 Oct 22;19(10):e0309988. doi: 10.1371/journal.pone.0309988 (PMC11495604; doi:10.1371/journal.pone.0309988)
Supplement: S1 File — (DOCX) [file pone.0309988.s001.docx]

**Supporting Information**

**S1 Table:** Raw data of the measurement of intracellular hydrogen peroxide production (μM) by whole cells as depicted in Figure 3.

| Bacterial strains/control | Time (min) | | | | | | |
| --- | --- | --- | --- | --- | --- | --- | --- |
|  | 0 | 7 | 14 | 21 | 28 | 35 | 42 |
| HPX^-^ | 0.1315 | 0.1708 | 0.1624 | 0.1618 | 0.1489 | 0.1450 | 0.1377 |
|  | 0.1731 | 0.1899 | 0.1910 | 0.1927 | 0.1776 | 0.1635 | 0.1472 |
|  | 0.1742 | 0.2439 | 0.2753 | 0.2927 | 0.3169 | 0.3192 | 0.3332 |
|  | 0.1922 | 0.2276 | 0.2596 | 0.2916 | 0.3349 | 0.3394 | 0.3298 |
|  | 0.0528 | 0.1173 | 0.1180 | 0.1298 | 0.1140 | 0.1035 | 0.0864 |
|  | 0.0495 | 0.0982 | 0.0890 | 0.0699 | 0.0699 | 0.0732 | 0.0555 |
| HPX^-^ *ΔfadE* | 0.1270 | 0.1629 | 0.1798 | 0.1686 | 0.1854 | 0.1804 | 0.1770 |
|  | 0.1399 | 0.1764 | 0.1815 | 0.1719 | 0.1770 | 0.1573 | 0.1579 |
|  | 0.2360 | 0.3253 | 0.3220 | 0.3568 | 0.3708 | 0.3866 | 0.4012 |
|  | 0.2040 | 0.2461 | 0.2911 | 0.3326 | 0.3708 | 0.3517 | 0.3720 |
|  | 0.1147 | 0.1443 | 0.1318 | 0.1410 | 0.1436 | 0.1423 | 0.0844 |
|  | 0.0291 | 0.0574 | 0.0660 | 0.0291 | 0.0739 | 0.0660 | 0.0495 |
| HPX^-^ *ΔfadR* | 0.1343 | 0.1629 | 0.2883 | 0.3647 | 0.3950 | 0.4551 | 0.4124 |
|  | 0.1017 | 0.1545 | 0.1601 | 0.1837 | 0.1967 | 0.2034 | 0.2130 |
|  | 0.2006 | 0.2641 | 0.2944 | 0.3017 | 0.3304 | 0.3714 | 0.3770 |
|  | 0.1585 | 0.2040 | 0.2573 | 0.2776 | 0.3096 | 0.3237 | 0.3293 |
|  | 0.0798 | 0.1311 | 0.1601 | 0.2009 | 0.2193 | 0.1923 | 0.2377 |
|  | 0.0647 | 0.1035 | 0.1772 | 0.2114 | 0.2621 | 0.2785 | 0.3160 |
| Media | 0.1478 | 0.1472 | 0.1365 | 0.2141 | 0.0955 | 0.1691 | 0.1427 |
|  | 0.0265 | -0.0064 | 0.0423 | 0.0252 | 0.0397 | 0.0199 | 0.0186 |
|  | 0.0014 | -0.0102 | -0.0016 | -0.0046 | -0.0105 | -0.0100 | -0.0122 |

**S2 Table:** Raw data for the growth representative of bacterial strains that exposed to the medium containing glycerol and dodecanoic acid as demonstrated in Figure 4A.

| Time (Hr) | MG1655 | HPX^-^ | HPX^-^*ΔfadE* | HPX^-^ *ΔfadR* | HPX^-^*ΔfadR ΔfadE* |
| --- | --- | --- | --- | --- | --- |
| 0 | 0.0124 | 0.0111 | 0.0108 | 0.011 | 0.0143 |
| 0.5 | 0.0143 | 0.012 | 0.011 | 0.0128 | 0.0138 |
| 1 | 0.0175 | 0.0155 | 0.0118 | 0.0145 | 0.0152 |
| 1.5 | 0.0226 | 0.0193 | 0.0129 | 0.0156 | 0.0184 |
| 2 | 0.033 | 0.0256 | 0.0155 | 0.0179 | 0.0227 |
| 2.5 | 0.0509 | 0.0347 | 0.0202 | 0.0202 | 0.0288 |
| 3 | 0.0835 | 0.0507 | 0.0297 | 0.0252 | 0.0396 |
| 3.5 | 0.1159 | 0.0675 | 0.0397 | 0.0298 | 0.0536 |
| 4 | 0.1906 | 0.0944 | 0.0587 | 0.04 | 0.072 |
| 4.5 | 0.2597 | 0.1274 | 0.0826 | 0.0519 | 0.0923 |
| 5 |  | 0.1743 | 0.1238 | 0.0663 | 0.1349 |
| 5.5 |  | 0.2308 | 0.1635 | 0.0845 | 0.1687 |
| 6 |  |  | 0.2228 | 0.1111 | 0.2239 |
| 6.5 |  |  |  | 0.1394 |  |
| 7 |  |  |  | 0.1744 |  |
| 7.5 |  |  |  | 0.2177 |  |

**S3 Table:** Raw data of the growth kinetics of the bacterial strains after adaptation to the medium as can be seen in Figure 4B.

| Time (Min.) | 0 | 15 | 30 | 45 | 60 | 75 | 90 | 105 | 120 |
| --- | --- | --- | --- | --- | --- | --- | --- | --- | --- |
| MG1655 | 0.0210 | 0.0270 | 0.0360 | 0.0490 | 0.0670 | 0.0850 | 0.1080 | 0.1380 | 0.1770 |
|  | 0.0210 | 0.0280 | 0.0360 | 0.0490 | 0.0680 | 0.0860 | 0.1090 | 0.1410 | 0.1790 |
|  | 0.0210 | 0.0280 | 0.0360 | 0.0490 | 0.0660 | 0.0860 | 0.1080 | 0.1380 | 0.1780 |
|  | 0.0220 | 0.0290 | 0.0370 | 0.0510 | 0.0710 | 0.0900 | 0.1130 | 0.1450 | 0.1890 |
| HPX^-^ | 0.0180 | 0.0210 | 0.0270 | 0.0360 | 0.0500 | 0.0640 | 0.0790 | 0.1010 | 0.1230 |
|  | 0.0180 | 0.0210 | 0.0280 | 0.0370 | 0.0500 | 0.0640 | 0.0790 | 0.1000 | 0.1350 |
|  | 0.0180 | 0.0220 | 0.0280 | 0.0380 | 0.0510 | 0.0670 | 0.0810 | 0.1030 | 0.1310 |
|  | 0.0190 | 0.0220 | 0.0290 | 0.0390 | 0.0530 | 0.0680 | 0.0840 | 0.1040 | 0.1360 |
| HPX^-^ *ΔfadR* | 0.0180 | 0.0220 | 0.0290 | 0.0360 | 0.0450 | 0.0570 | 0.0700 | 0.0860 | 0.1050 |
|  | 0.0190 | 0.0230 | 0.0290 | 0.0370 | 0.0460 | 0.0590 | 0.0740 | 0.0900 | 0.1090 |
|  | 0.0180 | 0.0220 | 0.0270 | 0.0350 | 0.0440 | 0.0560 | 0.0690 | 0.0850 | 0.1040 |
|  | 0.0190 | 0.0230 | 0.0290 | 0.0360 | 0.0450 | 0.0580 | 0.0720 | 0.0890 | 0.1080 |
| AL441 *ΔfadR* | 0.0220 | 0.0290 | 0.0390 | 0.0530 | 0.0700 | 0.0880 | 0.1110 | 0.1410 | 0.1810 |
|  | 0.0220 | 0.0290 | 0.0390 | 0.0530 | 0.0700 | 0.0890 | 0.1100 | 0.1400 | 0.1790 |
|  | 0.0150 | 0.0200 | 0.0280 | 0.0380 | 0.0510 | 0.0660 | 0.0840 | 0.1070 | 0.1350 |
|  | 0.0160 | 0.0220 | 0.0300 | 0.0400 | 0.0540 | 0.0700 | 0.0890 | 0.1140 | 0.1440 |

**S4 Table:** Raw data for β-Galactosidase reporter assay as shown in Figure 5.

| Bacterial strains |  | Glycerol | Glycerol + C12 | C12 |
| --- | --- | --- | --- | --- |
| AL441 | Mean | 310.779 | 264.6481 | 300.1334 |
|  | STD | 18.4689 | 8.7493 | 38.2037 |
|  | n | 5 | 5 | 5 |
| *ΔfadR* | Mean | 383.4366 | 292.8948 | 383.0384 |
|  | STD | 22.3919 | 23.6802 | 48.0182 |
|  | n | 5 | 5 | 5 |
| *ΔfadE* | Mean | 291.5271 | 268.0609 | 298.4268 |
|  | STD | 50.4898 | 3.5515 | 42.6883 |
|  | n | 5 | 5 | 5 |
| *ΔfadR ΔfadE* | Mean | 341.1802 | 241.0882 | 247.5839 |
|  | STD | 6.4359 | 19.8283 | 16.1681 |
|  | n | 5 | 5 | 5 |
| AL495 (HPX^-^) | Mean | 4738 |  |  |
|  | STD | 706 |  |  |
|  | n | 3 |  |  |

**S5 Table:** *In Vitro* reactivity of Dihydrolipoic acid (DHLA) with hydrogen peroxide, DTT was used as positive control for comparison as demonstrated in Figure 6.

| Time (min) | 0 μM DHLA | 15 μM DHLA | 60 μM DHLA | 150 μM DHLA |
| --- | --- | --- | --- | --- |
| 0 | 12.20 | 11.10 | 8.20 | 7.70 |
| 5 | 11.60 | 7.80 | 8.80 | 8.10 |
| 10 | 11.20 | 9.80 | 8.40 | 7.40 |
| 20 | 12.40 | 12.30 | 8.20 | 7.90 |
| 30 | 13.20 | 12.00 | 10.20 | 10.50 |
| 40 | 12.50 | 12.20 | 10.80 | 9.70 |

| Time (min) | 0 μM DTT | 60 μM DTT |
| --- | --- | --- |
| 0 | 12.40 | 7.90 |
| 5 | 12.40 | 7.30 |
| 10 | 12.70 | 6.40 |
| 15 | 12.60 | 5.70 |
| 20 | 13.00 | 5.20 |
| 30 | 13.10 | 4.50 |
| 40 | 12.80 | 3.80 |
