# Supplementary material for: Evidence for endogenous hydrogen peroxide production by E. coli fatty acyl-CoA dehydrogenase
Source: PLoS One. 2024 Oct 22;19(10):e0309988. doi: 10.1371/journal.pone.0309988 (PMC11495604; doi:10.1371/journal.pone.0309988)
Supplement: Attachment — Submitted filename: response to reviewers.docx [file pone.0309988.s002.docx]

Response to reviewers

Manuscript ID: PONE-D-24-26458

Reviewer's Comments 1:

Review comment on “Evidence for endogenous hydrogen peroxide production by *E. coli* fatty acyl-CoA dehydrogenase”

General comment: This is a nice piece of work on detail understanding on how superoxides and H_2_O_2_ are produced continuously in bacterial cells. Results presented in figures but not described elaborately, indeed results are given partially as in typical “results & Discussion” section, while this article has again a separate discussion section. So, it is actually better combine both sections as “Results and Discussion”, and describe a little of the Figures as a result component of the section.

Response: Thank you for your reading of our manuscript. As requested, we have combined the Results and Discussion sections.

Specific comment:

Lines22-23: “To avoid this problem cells maintain high levels of scavenging enzymes” provide names of a few important scavenging enzymes relevant to this study.

Response: the phase “scavenging enzymes” has been replaced with “superoxide dismutases, catalases, and peroxidases.”

Lines115-116: “We observed that when the dehydrogenase, encoded by FadE, is strongly expressed, it elevates the rate of cellular H2O2 production.” Is there any information or did you try the rate of cellular H2O2 production in absence of FadE?

Response: We did this experiment, as demonstrated in Figure 3. As expected, the absence of *fadE* has no impact on H_2_O_2_ formation in a *fadR^+^* strain, because the enzyme is not induced under those conditions. Induction—and high H_2_O_2_ production—occurs in the *fadR* mutant. Accordingly, Figure 4 confirms that the growth defect created by the *fadR* mutation is reversed when *fadE* is deleted.

Line130-131: What is the source of dodecanoic acid? What concentration of ethanol was used to prepare dodecanoic acid/ and at what ratio to the LB medium ethanol was used (what would be final ethanol concentration in LB growth medium)? For H2O2 production studies in various genetic backgrounds, why you did not use WT cells along with media as controls?

Response: The dodecanoic acid was purchased from Sigma and dissolved in absolute ethanol as a 50 mM stock solution before dilution into the working media. Thus, the final concentration of ethanol was 0.1 %, which is not toxic to the bacterium. This information has now been added to the Materials & Methods.

Wild-type cells (MG1655) do not release any H_2_O_2_, and we reproduced this result. This observation is now stated in the section of the Materials & Methods section that presents the rationale for doing these experiments in an HPX^-^ (scavenging-deficient) background.

Table1: write the full form of NEB. NO need to write “REF” just numbering is enough. Check for accurateness of the “Reference” column, which should have appropriate references/sources only.

Response: Done.

Figure 6 legend, provide full forms of DTT and DHLA.

Response: Done.

Reviewer's Comments 2:

MS # PONE-D-24-26458 intends to identify major site of ROS generation, which is ubiquitously loosely attributed to FADH2 oxidation during aerobic respiration. However, there are many oxidative pathways wherein FADH2 generates and enters the oxidative electron transport system for ATP production and electron leakage responsible for ROS generation. With the help of E. coli fadE (Acyl-CoA dehydrogenase) mutant, the generation of hydrogen peroxide has been monitored during the in vitro βoxidation of dodecanoic acid, a fatty acid, for the mutant was incapable of scavenging the generated hydrogen peroxide. The authors are inclined to believe that βoxidation generates the major source of ROS within the limits of their experimental setup. Of course, the findings are significant and prompts the further advanced work.

Experimental design and data generation is sound and appears to be reproducible. Results and Discussion are posited logically.

However, the MS needs through scrutiny for standard usage of English language e.g., use “must arise” in place of “must be arise” in Line 45-46 “However, the investigators quickly inferred that these oxygen species must be arise inside cells and be capable of damaging the organism.”

Response: Done.
